# Supplementary material for: The Effect of Leaf Traits on the Excitation, Transmission, and Perception of Vibrational Mating Signals in the Tea Leafhopper Empoasca onukii Matsuda (Hemiptera: Cicadellidae)
Source: Plants (Basel). 2025 Apr 7;14(7):1147. doi: 10.3390/plants14071147 (PMC11991016; doi:10.3390/plants14071147)
Supplement: Supplementary file 1 [file plants-14-01147-s001.zip › Figure S1.pdf]

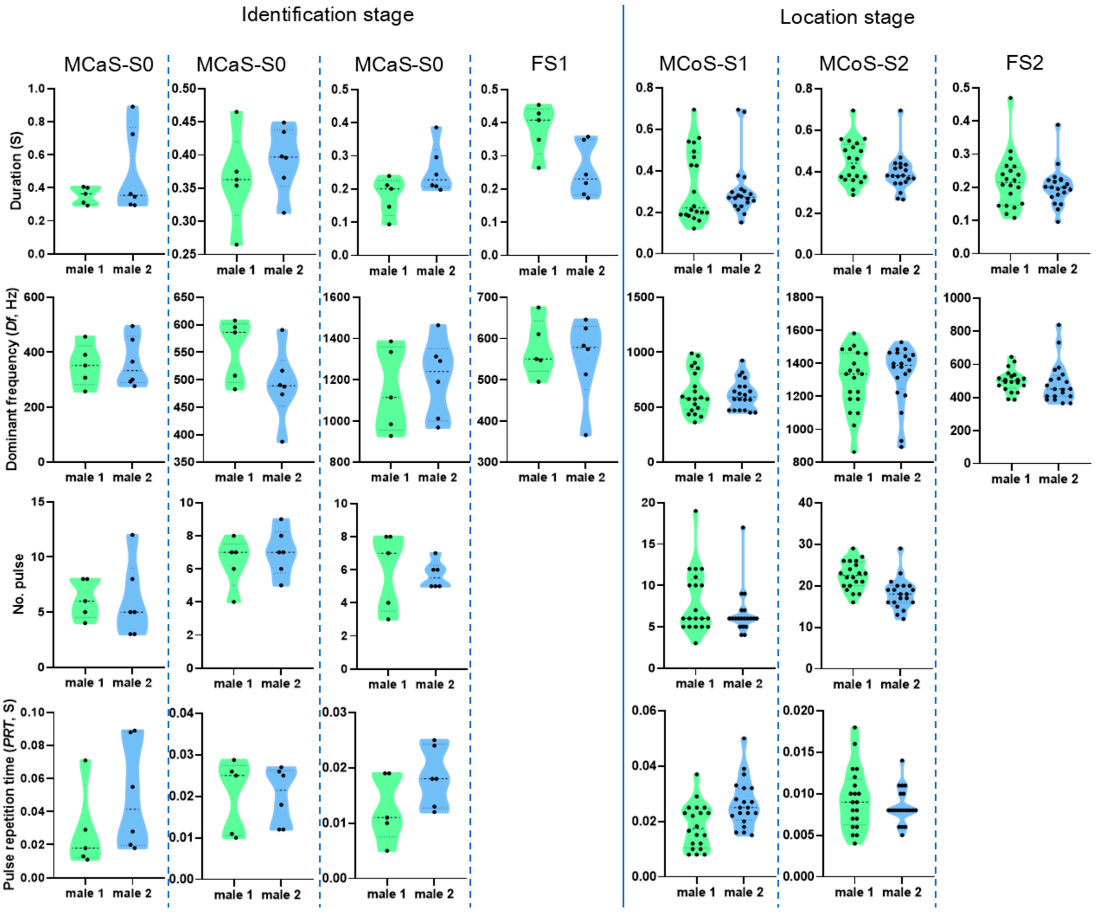

Figure S1 Intraindividual variability in the mating signal parameters of *Empoasca onukii*. The parameters of mating signals derived from two different pairs on the seventh leaf under the bud in 4.4.2, including all the identification duets and 20 randomly selected location duets in each signal sample. The signal parameters of an *E. onukii* individual were variable within a certain range.
