# Supplementary material for: The Effect of Leaf Traits on the Excitation, Transmission, and Perception of Vibrational Mating Signals in the Tea Leafhopper Empoasca onukii Matsuda (Hemiptera: Cicadellidae)
Source: Plants (Basel). 2025 Apr 7;14(7):1147. doi: 10.3390/plants14071147 (PMC11991016; doi:10.3390/plants14071147)
Supplement: Supplementary file 1 [file plants-14-01147-s001.zip › Table S1.pdf]

Table S1 Analysis of variance of the parameters of each section of MCaSs after transmission to the 7th leaf below the tea bud.

Test of Homogeneity of Variance					
MCaS section	Parameter	Levene Statistic	df_1	df_2	*Sig.*
S0	*Df*	1.53	5	114	0.19
	Duration	1.61	5	114	0.16
	I	13.49	5	114	<0.001
S1	*Df*	2.91	5	114	0.02
	Duration	1.70	5	114	0.14
	I	13.75	5	114	<0.001
S2	*Df*	10.43	5	114	<0.001
	Duration	1.54	5	114	0.18
	I	7.21	5	114	<0.001
ANOVA					
MCaS section	Parameters	*F*/*Welch*	df_1	df_2	*Sig.*
S0	*Df*	0.36	5	114	0.87
	Duration	1.21	5	114	0.31
	I	8.22	5	51.79	<0.001
S1	*Df*	2.04	5	114	0.07
	Duration	2.05	5	114	0.08
	I	10.44	5	51.58	<0.001
S2	*Df*	1.54	5	52.29	0.19
	Duration	5.09	5	114	<0.001
	I	9.97	5	51.46	<0.001

Using leaf age as the categorical variable, each signal parameter of the transmitted MCaSs was compared by analysis of variance. One-way ANOVA was used when the data met assumptions of normality and homoscedasticity; otherwise, Welch's ANOVA was used ($P < 0.05$). Abbreviations of signal parameters are shown in Table 1.
