# Supplementary material for: The Effect of Leaf Traits on the Excitation, Transmission, and Perception of Vibrational Mating Signals in the Tea Leafhopper Empoasca onukii Matsuda (Hemiptera: Cicadellidae)
Source: Plants (Basel). 2025 Apr 7;14(7):1147. doi: 10.3390/plants14071147 (PMC11991016; doi:10.3390/plants14071147)
Supplement: Supplementary file 1 [file plants-14-01147-s001.zip › Table S2.pdf]

Table S2 Comparison of MCaS parameters in female responses and non-responses

Section	Parameters		Levene's test		*t* test		
			F	*Sig.*	*t*	*df*	Sig.
S0	*Df*	Equal variance assumed	1.06	0.31	-0.44	118.00	0.66
		Equal variance not assumed			-0.44	117.87	0.66
	duration	Equal variance assumed	0.43	0.52	1.96	118.00	0.05
		Equal variance not assumed			1.98	117.90	0.05
	I	Equal variance assumed	1.88	0.17	-0.83	118.00	0.41
		Equal variance not assumed			-0.86	113.72	0.39
S1	*Df*	Equal variance assumed	0.44	0.51	-0.17	118.00	0.86
		Equal variance not assumed			-0.17	108.27	0.87
	duration	Equal variance assumed	1.94	0.17	-1.22	118.00	0.22
		Equal variance not assumed			-1.25	116.87	0.21
	I	Equal variance assumed	0.49	0.49	0.42	118.00	0.68
		Equal variance not assumed			0.42	110.05	0.68
S2	*Df*	Equal variance assumed	6.65	0.01	0.98	118.00	0.33
		Equal variance not assumed			0.96	101.17	0.34
	duration	Equal variance assumed	3.64	0.06	-1.09	118.00	0.28
		Equal variance not assumed			-1.12	115.48	0.27
	I	Equal variance assumed	2.51	0.12	0.89	118.00	0.37
		Equal variance not assumed			0.86	88.38	0.39

In test 4.4.1, MCaS parameters from the two categories (female responses and non-responses) were compared by an independent sample *t*-test (two-tailed, $P < 0.05$; SPSS 25.0). Abbreviations of signal parameters are shown in Table 1.
