# Supplementary material for: The Effect of Leaf Traits on the Excitation, Transmission, and Perception of Vibrational Mating Signals in the Tea Leafhopper Empoasca onukii Matsuda (Hemiptera: Cicadellidae)
Source: Plants (Basel). 2025 Apr 7;14(7):1147. doi: 10.3390/plants14071147 (PMC11991016; doi:10.3390/plants14071147)
Supplement: Supplementary file 1 [file plants-14-01147-s001.zip › Table S3.pdf]

Table S3 Analysis of variance of parameters in each section of the MCaS and FS1 and behavioral indexes in the identification stage.

Test of Homogeneity of Variance					
Parameter/index		Levene Statistic	df_1	df_2	*Sig.*
MCaS-S0	*Df*	1.85	9	60	0.061
	N_{pulse}	4.80	9	60	<0.001
	PRT	2.54	9	60	0.01
	duration	1.39	9	60	0.2
MCaS-S1	*Df*	5.92	9	60	<0.001
	N_{pulse}	2.50	9	60	0.01
	PRT	2.90	9	60	0.0031
	duration	1.68	9	60	0.1
MCaS-S2	*Df*	1.48	9	60	0.158204
	N_{pulse}	3.02	9	60	0.002
	PRT	5.35	9	60	<0.001
	duration	2.56	9	60	0.008
FS1	*Df*	1.30	9	60	0.24
	duration	1.95	9	60	0.047
Female behavior	Delay	7.87	9	60	<0.001
ANOVA					
Parameter/index		*F*	df_1	df_2	*Sig.*
MCaS-S0	*Df*	3.84	9	35.31	<0.001
	N_{pulse}	2.68	9	35.72	0.01
	PRT	1.49	9	35.36	0.17
	duration	0.82	9	60.00	0.60
MCaS-S1	*Df*	2.88	9	35.48	0.01
	N_{pulse}	2.65	9	34.40	0.01
	PRT	1.83	9	34.70	0.08
	duration	2.23	9	60.00	0.02
MCaS-S2	*Df*	1.34	9	60.00	0.22
	N_{pulse}	1.53	9	34.41	0.15
	PRT	2.64	9	35.59	0.01
	duration	0.81	9	34.39	0.61
FS1	*Df*	1.24	9	60.00	0.27
	duration	5.77	9	32.77	<0.001
Female behavior	Delay	3.17	9	34.35	0.0027

Using leaf age as the categorical variable, each signal parameter or female behavioral index was compared by analysis of variance. One-way ANOVA was used when the data met assumptions of normality and homoscedasticity; otherwise, Welch's ANOVA was used ($P < 0.05$). Abbreviations of signal parameters are shown in Table 1.
