# Supplementary material for: The Effect of Leaf Traits on the Excitation, Transmission, and Perception of Vibrational Mating Signals in the Tea Leafhopper Empoasca onukii Matsuda (Hemiptera: Cicadellidae)
Source: Plants (Basel). 2025 Apr 7;14(7):1147. doi: 10.3390/plants14071147 (PMC11991016; doi:10.3390/plants14071147)
Supplement: Supplementary file 1 [file plants-14-01147-s001.zip › Table S4.pdf]

Table S4 Analysis of variance of parameters of each section of the MCoS and FS2 and behavioral indexes in the localization stage.

Test of Homogeneity of Variance					
Parameter/index		Levene Statistic	df_1	df_2	*Sig.*
MCoS-S1	*Df*	11.04	9	60	<0.001
	N_{pulse}	2.52	9	60	0.008
	PRT	3.63	9	60	<0.001
	duration	3.05	9	60	0.001
MCoS-S2	*Df*	0.98	9	60	0.457
	N_{pulse}	5.19	9	60	<0.001
	PRT	2.83	9	60	0.003
	duration	5.82	9	60	<0.001
FS2	*Df*	1.95	9	60	0.043
	duration	1.80	9	60	0.066
Female behavior	Delay	6.41	9	60	<0.001
Male behavior	Interval	3.72	9	60	<0.001
	$t_{courtship}$	5.75	9	60	<0.001
	N_{cycle}	3.08	9	60	0.004
ANOVA					
Parameter/index		*F*	df_1	df_2	*Sig.*
MCoS-S1	*Df*	3.81	9	39.34	<0.001
	N_{pulse}	1.73	9	40.08	0.084
	PRT	1.10	9	38.92	0.365
	duration	4.25	9	39.29	<0.001
MCoS-S2	*Df*	1.73	9	60.00	0.079
	N_{pulse}	5.57	9	39.80	<0.001
	PRT	2.61	9	38.73	0.007
	duration	5.04	9	39.25	<0.001
FS2	*Df*	1.24	9	60.00	0.274
	duration	5.77	9	34.77	<0.001
Female behavior	Delay	17.76	9	37.69	<0.001
Male behavior	Interval	5.92	9	38.30	<0.001
	$t_{courtship}$	0.95	9	25.76	0.503
	N_{cycle}	1.15	9	25.17	0.369

Using leaf age as the categorical variable, each signal parameter or behavioral index was compared by analysis of variance. One-way ANOVA was used when the data met assumptions of normality and homoscedasticity; otherwise, Welch's ANOVA was used ($P < 0.05$). Abbreviations of signal parameters are shown in Table 1.
